# Associative pathways of school bullying with adolescent internet addiction and the moderating role of emotional resilience

**DOI:** 10.3389/fpsyt.2026.1836080

**Published:** 2026-06-09

**Authors:** Xinyi Pang, Xinfeng Zhang, Chengguo Peng, Zheming Tu

**Affiliations:** 1 Jingzhou Hospital Affiliated to Yangtze University, Jingzhou, Hubei, China; 2 Mental Health Institute of Yangtze University, Jingzhou, China

**Keywords:** adolescents, school bullying, anxiety, depression, insomnia, internet addiction, emotional resilience

## Abstract

**Objective:**

This study explores the associative pathways between school bullying and adolescent internet addiction among middle school students in Jingzhou City, as well as the potential associative pattern of emotional resilience. It aims to clarify the psychological correlation pathways underlying the association between school bullying and internet addiction, examine the multivariate correlational pattern of these variables, and further discuss the practical value of emotional resilience for targeted preventive intervention.

**Methods:**

This study recruited 19, 601 adolescents from 22 middle schools in Jingzhou District in 2025 using a cluster sampling design. Data were collected using a self-designed general demographic questionnaire together with standardized scales, including the School Bullying Scale, Internet Addiction Test (IAT), Generalized Anxiety Disorder-7 (GAD-7), Patient Health Questionnaire-9 (PHQ-9), Insomnia Severity Index (ISI), and Emotional Resilience Questionnaire (ERQ). All data were analyzed using SPSS 27.0 and Process 5.0 macro program, adopting descriptive statistics, correlation analysis, multicollinearity test, regression analysis, mediation and moderation analysis, as well as robustness analysis.

**Results:**

A total of 20, 225 questionnaires were distributed, and 19, 601 valid responses were finally included, yielding an effective response rate of 96.91%. Significant positive correlations were observed between school bullying and adolescent internet addiction. Serial and independent indirect associative pathways were identified: the serial pathway of school bullying→anxiety→depression→internet addiction, and two independent indirect links of school bullying→anxiety→internet addiction and school bullying→depression→internet addiction. Emotional resilience showed a moderating correlation in such pathways, with higher emotional resilience linked to lower levels of anxiety and depression, and further correlated with a lower tendency toward internet addiction. Robustness analyses supported the stability of these observed associative patterns.

**Conclusion:**

School bullying is closely correlated with anxiety, depression, insomnia and internet addiction in adolescents. Emotional resilience is negatively correlated with these negative emotional and behavioral outcomes. This study clarifies the statistical associative pathways between school bullying and adolescent internet addiction, and indicates that higher emotional resilience is correlated with a weaker associative tendency between school bullying and internet addiction. All findings are supported by robustness checks.

## 1 Introduction

School bullying has long been a major public health issue for adolescents, drawing extensive public attention. The United Nations Educational, Scientific and Cultural Organization (UNESCO) defines school bullying as aggressive behaviors occurring between school-aged children that go against the free will of those who are targeted. Characterized by a clear power imbalance, such behaviors tend to happen repeatedly over time and include four common types: physical, verbal, relational/social, and cyberbullying (1, 2). Given that various forms of bullying victimization often co-occur and share similar psychological impacts on adolescents, this study focuses on general school bullying victimization rather than specific subtypes or bullying perpetration. The 2018 global statistics on school bullying published by the UNESCO Institute for Statistics (UIS) indicate that roughly one out of every three adolescents worldwide have experienced school bullying, and this issue is still prevalent in countries of all income levels (3); As for the domestic context, the actual occurrence of school bullying in China is characterized by the coexistence of open bullying and nascent hidden bullying. A national investigation shows that the prevalence of repeated bullying victim experiences among primary and middle school students in China over the past year stands at 13.9%, and another 23.9% of these students have experienced mental distress because of others’ so-called “pranks”, a form of conduct referred to as initial bullying (4).

Adolescence represents a critical developmental transition between childhood and adulthood, involving rapid psychological, biological, and emotional maturation. Compared with college students or adults, they are especially vulnerable to bullying, which may lead to not only anxiety and depression but also but also conduct problems, oppositional defiant symptoms, and even antisocial personality traits and substance dependence in adulthood (4, 5). Negative experiences during this period can shape lifelong developmental trajectories, justifying the focus on adolescents as a high-priority group. During this critical developmental period, it can cause lasting impairments, underscoring the need to identify modifiable risk factors such as bullying.

When teenagers encounter school bullying, they are much more likely to develop negative affective symptoms such as anxiety and depression, and these symptoms are often accompanied by sleep disorders like insomnia. These unfavorable conditions push them to seek an escape from real-life stress through the internet, ultimately leading them to develop internet addiction. From established theoretical perspectives, bullied adolescents often lack peer support and social capital at school, leaving them less able to cope with stress and more prone to emotional distress (6–8). According to the social compensation hypothesis, due to impaired social functions in real life—such as poor friendship quality caused by bullying—individuals tend to compensate for their social attachment needs or desire for closeness through virtual interaction, which makes adolescents more vulnerable to digital device dependence or internet addiction (9). To alleviate negative emotions and compensate for unmet social needs in real life, they may engage in excessive internet use as a coping strategy, which increases the risk of internet addiction (10–13). Consistent with the Pathway Model of Problematic Mobile Phone Use and the I-PACE model, adolescents who experience bullying may develop emotional distress such as anxiety and depression, which in turn increases their risk of internet addiction (11, 14). Anxiety and depression are common emotional disorders, and they’re well established as key mediating factors that connect traumatic life experiences to addictive behaviors. Insomnia undermines individuals’ emotional regulation capacities, and this impairment can in turn further worsen the progression of internet addiction. Meanwhile, emotional resilience is a fundamental psychological resource that helps people cope with adversity, and it can ease the harmful associations with school bullying. Grounded in the conceptual framework by Davidson et al., emotional resilience enables adolescents to recover rapidly from stressful experiences such as bullying. Adolescents with higher emotional resilience are less likely to escape into internet use to relieve negative emotions, thus lowering their vulnerability to internet addiction (15, 16). People with higher levels of emotional resilience are more proficient at managing negative emotions and reducing their excessive reliance on the internet. Both bullying victimization and perpetration are linked to emotional problems and addictive behaviors (5, 17), and this study focuses exclusively on bullying victimization rather than bullying perpetration.

Despite these existing findings, the serial and sequential mechanisms linking school bullying to internet addiction remain unclear. Specifically, how anxiety, depression, and insomnia act as sequential mediators in this pathway has not been fully elucidated. Although prior work has examined simple mediation paths (18, 19), the integrated serial mediating pathway of anxiety → depression → insomnia has rarely been examined. Furthermore, the combined moderating role of emotional resilience in this serial chain remains underexplored, representing a critical research gap.

Based on these above-mentioned research results, this study constructs and verifies a moderated serial mediation model to address these gaps, which is used to clarify the way that school bullying is associated with internet addiction through a sequence of emotional problems including depression, anxiety and insomnia. Emotional resilience is also added as a moderating variable in the model to test its buffering role in the entire serial mediation process. This study sets out to explore the intrinsic mechanisms that underlie the relationship between school bullying and adolescent internet addiction, with the goal of providing empirical support for targeted interventions.

## 2 Methods

### 2.1 Study participants

A cluster sampling method was used to recruit middle school students from 22 middle schools in Jingzhou City in 2025. The age of participants ranged from 8 to 20 years based on the actual student population included in the cluster sampling. Inclusion criteria: (1) any gender;(2) native Chinese speakers with basic reading comprehension ability to independently understand all questionnaire items in Chinese;(3) providing written informed consent. Exclusion criteria: (1) severe mental or physical illness preventing questionnaire completion; (2) recent major life events. Head teachers were responsible for reporting students who were unable to independently understand or complete the questionnaires for any reason, and these students were excluded subsequently. Data were collected via the Wenjuanxing platform during computer classes or at home using parents’ mobile devices. A total of 20, 225 questionnaires were collected; 19, 601 valid questionnaires were included in analyses, with an effective response rate of 96.91%.Each student was assigned a unique ID, which can distinguish different students across schools and classes, ensure data validity, and allow longitudinal comparison with follow-up data from the same individuals. Written informed consent was obtained from all participants. For participants under 18 years of age, additional written informed consent was obtained from their parents or legal guardians. Intraclass correlation coefficients (ICC) were computed for all core variables across the all schools to assess potential school-level clustering (20). All ICC values were 0, indicating no hierarchical nesting or clustering effects in the data. This study was approved by the Ethics Committee of Jingzhou Mental Health Center. (Serial Number 2021LL0501).

### 2.2 Research tools

All instruments in this study were adopted the validated Mandarin Chinese version. These translated scales have been widely used in domestic adolescent research, with plain and culturally adapted item wording that is suitable for participants in the current study.

#### 2.2.1 Demographic variables include gender, grade, household registration, only-child status and family economic status

#### 2.2.2 School bullying scale

All scales in this study were adopted the validated Mandarin Chinese version. The Chinese Adolescent School Bullying Behavior Scale (Sun & Liu, 2022) was used as the localized assessment tool for school bullying in this study (21). Six items were selected from the scale, which was scored on a 4-point scale ranging from 1 to 4 (1 = never, 4 = once a week or more frequently). The higher the total score, the greater the frequency and severity of bullying victimization an individual has experienced; total scores ranging from 7 to 24 indicate the presence of school bullying victimization in an individual. In the present study, the Cronbach’s α coefficient of the full scale was 0.861, and the α coefficients for its subscales were 0.727 for physical bullying, 0.579 for verbal bullying and 0.713 for relational bullying, respectively, and the McDonald’s omega coefficient was 0.867.

#### 2.2.3 Generalized anxiety disorder-7 scale

This scale was implemented with the validated Mandarin Chinese version. The scale developed by Spitzer et al. (2006), assesses symptoms of generalized anxiety disorder (22). It consists of seven core items, each rated on a 4−point Likert−type scale (0 = not at all, 3 = nearly every day). Higher total scores indicate more severe anxiety symptoms. In this study, the scale demonstrated good internal consistency, with a Cronbach’s α of 0.955, and the McDonald’s omega coefficient was 0.955.

#### 2.2.4 Patient health questionnaire-9 scale

This study used the validated Mandarin Chinese version of the scale. Developed by Spitzer et al.(2001), the PHQ-9 is widely used to screen for depressive symptoms and assess their severity (23). It consists of 9 core items and employs a 1–4 rating scale (1=Not at all, 4=Almost always). The higher the total score, the more severe the individual’s depressive symptoms; a total score ranging from 5 to 27 indicates the presence of depressive problems in the individual. In the present study, the Cronbach’s α was 0.936, which indicates that its internal consistency reliability meets psychometric standards, and the McDonald’s omega coefficient was 0.937.

#### 2.2.5 Insomnia severity index

Developed by Morin (1993), the Insomnia Severity Index (ISI) was applied with its validated Mandarin Chinese version in the current study. It comprises 7 items and adopts a 0–4 rating scale (0 = No difficulty, 4 = Extremely severe difficulty). The higher the total score, the greater the severity of an individual’s insomnia; a total score ranging from 8 to 28 indicates the presence of insomnia problems in the individual (24). In this study, the scale demonstrated stable internal consistency, with a Cronbach’s α of 0.892, and the McDonald’s omega coefficient was 0.891.

#### 2.2.6 Internet addiction test

The IAT is a unidimensional self-report scale adapted and extended from Young’s Diagnostic Questionnaire (YDQ) for Internet addiction (25). The validated Mandarin Chinese version was adopted in this study. It contains 20 items rated on a 5-point Likert scale (1 = rarely, 5 = always). Higher total scores indicate more severe levels of Internet addiction, with a clinical cutoff score ranging from 50 to 100 suggesting problematic use. In the present study, the scale demonstrated good internal consistency, with a Cronbach’s α coefficient of 0.961, and the McDonald’s omega coefficient was 0.941.

#### 2.2.7 Emotional resilience questionnaire

The scale used in the present study was adapted from the conceptual framework proposed by Davidson et al., and was later revised by Chinese scholars to be applicable to adolescent populations (26, 27). It comprises 11 items assessed on a 6-point Likert scale (1=strongly disagree, 6=strongly agree). Higher total scores reflect a greater ability to positively transform negative emotions, indicating stronger emotional resilience. In the present sample, the scale exhibited satisfactory internal consistency, with a Cronbach’s α coefficient of 0.876, and the McDonald’s omega coefficient was 0.843.

### 2.4 Data processing

This study used SPSS 27.0 and the PROCESS 5.0 macro for data analysis, including descriptive statistics and correlation analysis. Harman’s single-factor test was conducted to evaluate common method bias. Prior to formal analysis, systematic data screening was conducted. The Kolmogorov–Smirnov (K-S) test, which is robust for large sample sizes, was applied to examine the univariate normality of all core variables. The results showed that all variables including GAD-7, PHQ-9, ISI, school bullying, emotional resilience and internet addiction significantly deviated from the normal distribution (all p < 0.001). Multivariate outliers were detected based on Mahalanobis distance and Cook’s distance, and no influential outlier or obvious floor and ceiling effects were found in this dataset. Descriptive statistics and correlation coefficients were calculated for demographic characteristics and core research variables. Multicollinearity diagnostics verified that no severe multicollinearity existed among variables; all primary variables were standardized and mean-centered before subsequent analyses. The 95% percentile bootstrap confidence intervals (CIs) with 5, 000 resamples were used to test mediation models. An effect was considered statistically significant at the 0.05 level when the 95% bootstrap CI did not contain zero. To test the research hypotheses, Model 6 and Model 85 in Hayes’ PROCESS macro were adopted to examine chained mediation and moderated mediation effects, respectively. Robustness checks were performed by replacing core measurement scales and adjusting the sequential order of mediating variables in the chained mediation model. Gender, grade, household registration (urban/rural), only-child status, and family socioeconomic status were incorporated as covariates in all analyses.

## 3 Results

### 3.1 Common method bias test

Common method bias was evaluated via Harman’s single-factor test. All statistical analyses in the present study adopted the maximum likelihood estimation method. All scale items were subjected to an exploratory factor analysis (EFA).Results indicated that six factors with eigenvalues greater than 1 were extracted from the unrotated factor solution, with the first factor explaining 20.038% of the total variance—which is below the critical threshold of 40%. This demonstrates that no serious common method bias existed in this study, thereby supporting the reliability of the data (28).

### 3.2 Descriptive statistics and multicollinearity analysis

#### 3.2.1 Descriptive statistics

Among the R valid questionnaires collected, adolescents with and without internet addiction demonstrated statistically significant differences (P < 0.05) in gender, grade, household registration (urban/rural), only-child status, and family economic level. See **Table 1** for detailed results.

**Table 1:** Descriptive statistics of relevant variables (N=19601).

Variable		Internet addiction		X^2^	P	Mean ± SD
		Yes	No (%)			
Gender	male	2871 (28.1)	7329 (71.9)	50.276	<0.001	–
	female	2228 (23.7)	7173 (76.3)			
Grade	Seventh grade	807 (17.1)	3925 (82.9)	444.941	<0.001	–
	Eighth grade	1012 (23.6)	3282 (76.4)			
	Ninth Grade	914 (25.4)	2680 (74.6)			
	Freshman year	991 (35.2)	1821 (64.8)			
	11th grade	748 (34.3)	1434 (65.7)			
	Senior Year	627 (68.4)	1360 (31.6)			
Household registration	rural household registration	3340 (27.7)	8721 (72.3)	45.901	<0.001	–
	non-rural household registration	1759 (23.3)	5781 (76.7)			
Only child	yes	2451 (25.3)	7221 (74.7)	4.490	0.034	–
	no	2648 (26.7)	7281 (73.3)			
Economic level	below average	792 (37.4)	1323 (62.6)	161.166	<0.001	–
	average level	4078 (24.6)	12499 (75.4)			
	above average	229 (25.2)	680 (74.8)			
School bullying	no	3197 (21.1)	11989 (78.9)	862.371	<0.001	6.93 ± 2.455
	yes	1902 (43.1)	3197 (56.9)			
Anxiety(GAD-7)	no	1808 (15)	10231 (85)	1960.444	0.000	4.15 ± 5.085
	yes	3291 (43.5)	4271 (56.5)			
Depression(PHQ-9)	no	1815 (14.7)	10545 (85.3)	2231.332	0.000	4.49 ± 5.779
	yes	3284 (45.4)	3957 (54.6)			
Insomnia(ISI)	no	2755 (18.7)	11957 (81.3)	1627.656	0.000	4.80 ± 5.016
	yes	2344 (47.9)	2545 (52.1)			

#### 3.2.2 Multicollinearity analysis

To further verify the presence of multicollinearity in the models, multicollinearity diagnostics were conducted. Multicollinearity is considered to exist when the tolerance value is below 0.1 and the Variance Inflation Factor (VIF) is greater than 10 (29). The VIF assesses the degree to which a single variable affects the standard error of regression estimates. The core variables included in the chained mediation and moderated mediation models (e.g., anxiety, depression, insomnia, school bullying, emotional resilience, internet addiction) as well as the covariates (gender, grade, household registration, only-child status, family economic level) were tested for multicollinearity using VIF and tolerance as diagnostic indices. The results indicated that the VIF values of all variables were less than 5, and the tolerance values were greater than 0.2, which were far lower than the critical thresholds for multicollinearity (VIF > 10, tolerance < 0.1). This suggests that no severe multicollinearity exists among the variables in the models, and such non-collinearity will not interfere with the parameter estimation of mediation and moderated mediation effects, thus ensuring the reliability and validity of the subsequent statistical analyses.

### 3.3 Correlation analysis

The Pearson correlation coefficient ranges from −1 to 1. This range defines the direction of the linear association between two variables (positive or negative correlation) and directly quantifies the strength of the linear relationship. As shown in **Table 2**, school bullying is significantly positively correlated with anxiety, depression, insomnia and Internet addiction, while exhibiting a significant negative correlation with emotional resilience. Anxiety is significantly positively correlated with depression, insomnia and Internet addiction, and negatively correlated with emotional resilience. Depression is significantly positively correlated with insomnia and Internet addiction, yet shows a significant negative correlation with emotional resilience. Insomnia is significantly positively correlated with internet addiction and negatively correlated with emotional resilience. Internet addiction is likewise significantly negatively correlated with emotional resilience. All the aforementioned correlations are statistically significant at p < 0.01.

**Table 2:** Correlation analysis of school bullying, anxiety, depression, insomnia, internet addiction and emotional resilience.

	1	2	3	4	5	6
1.School bullying	1					
2.Anxiety(GAD-7)	.294**	1				
3.Depression(PHQ-9)	.320**	.868**	1			
4.Insomnia(ISI)	.288**	.636**	.703**	1		
5.Internet addiction(IAT)	.248**	.437**	.467**	.410**	1	
6.Emotional resilience(ERQ)	-.206**	-.579**	-.567**	-.521**	-.435**	1

** indicates significance at the 0.01 level (two-tailed).

### 3.4 Results of chained mediation effect testing

This study employed SPSS 27.0 and the PROCESS 5.0 macro (Model 6) to examine the chained mediation effect among school bullying (X), anxiety (M1), depression (M2), insomnia (M3), and internet addiction (Y). Prior to the analysis, all core variables were preliminarily standardized and mean-centered. Incorporating seven control variables (e.g., gender and grade), a chained mediation model was established based on Pearson correlation analysis to validate the multi-path chained mediation mechanism. The bootstrap method (n = 5000) was applied to adjust standard errors, with bias-corrected confidence intervals used to assess the significance of the mediation effect. Specifically, a mediation effect is considered non-significant when its 95% confidence interval contains 0, and significant when it does not.

**Table 3** shows the statistic result: It is evident that the total effect of school bullying on adolescent internet addiction was statistically significant. The direct effect between school bullying and internet addiction was also statistically significant, accounting for 38% of the total effect. This indicates that school bullying is associated with adolescent internet addiction through both direct and indirect pathways involving multiple mediators. The total indirect effect was also statistically significant and substantial, accounting for 62% of the total effect, supporting the overall reasonableness of the proposed serial mediation model. All seven specific indirect pathways were statistically significant, as confirmed by 95% bootstrap confidence intervals that did not include zero. The specific results are as follows: ① The pathway school bullying → anxiety → internet addiction had a mediating effect size of 0.2246, accounting for 16% of the total effect; ② The pathway school bullying → depression → internet addiction had a mediating effect size of 0.0963, accounting for 7% of the total effect; ③ The pathway school bullying → insomnia → internet addiction had a mediating effect size of 0.0538, accounting for 4% of the total effect.

**Table 3:** Serial mediation effects of school bullying, anxiety, depression, insomnia and internet addiction (model 6).

Intermediary path	Effect	BootSE	BootLLCI	BootULCI	Significance	Proportion mediated
Total effect	1.4281	0.0404	1.3489	1.5072	yes	100%
Direct effect	0.5485	0.0388	0.4725	0.6246	yes	38%
Total indirect effect	0.8795	0.0314	0.8193	0.942	yes	62%
1. X→M1→Y	0.2246	0.0256	0.175	0.2752	yes	16%
2. X→M2→Y	0.0963	0.0093	0.079	0.1151	yes	7%
3. X→M3→Y	0.0538	0.0074	0.0396	0.0689	yes	4%
4. X→M1→M2→Y	0.3416	0.0269	0.2896	0.3954	yes	24%
5. X→M1→M3→Y	0.0227	0.0036	0.0161	0.0301	yes	2%
6. X→M2→M3→Y	0.0309	0.0034	0.0244	0.0377	yes	2%
7. X→M1→M2→M3→Y	0.1096	0.0100	0.0900	0.1294	yes	8%

X, Mean-centered school bullying; M1, Mean-centered anxiety; M2, Mean-centered depression; M3, Mean-centered insomnia; Y, Mean-centered internet addiction.

Among all the chain mediation routes, the path of school bullying→anxiety→depression→internet addiction turned out to be the most significant indirect path, accounting for 24% of the overall effect and therefore acting as the core associative pathway. The paths of school bullying→anxiety→insomnia→internet addiction and school bullying→depression→insomnia→internet addiction showed effect sizes of 0.0227 and 0.0309 respectively, and each of them accounts for 2% of the overall effect. Lastly, the full chain path of school bullying→anxiety→depression→insomnia→internet addiction presented an effect size of 0.1096, making up 8% of the overall effect. When school bullying is viewed as a stressor, the temporal sequence where anxiety emerges prior to depression is repeatedly confirmed by empirical evidence from many longitudinal studies, and it is also supported by existing empirical evidence (30, 31). Taken together, school bullying is indirectly associated with Internet addiction via seven distinct pathways, encompassing single mediation, dual chained mediation, and triple chained mediation. Collectively, these pathways form a complex and statistically significant associative mediating framework.

### 3.5 Results of moderated mediation effect testing

To examine the moderated mediation effect of emotional resilience (moderating variable W) on the pathway whereby school bullying (X) influences internet addiction (Y) through anxiety (M1), depression (M2), and insomnia (M3), all core variables were first subjected to mean-centering pretreatment in this study. SPSS 27.0 and the PROCESS 5.0 macro (Model 85) were adopted for analysis. Hierarchical regression was performed via SPSS to test the significance of the main effects and interaction terms, and Bootstrap sampling with 5000 resamples was applied to verify the moderated mediation effect.

**Figure 1:**
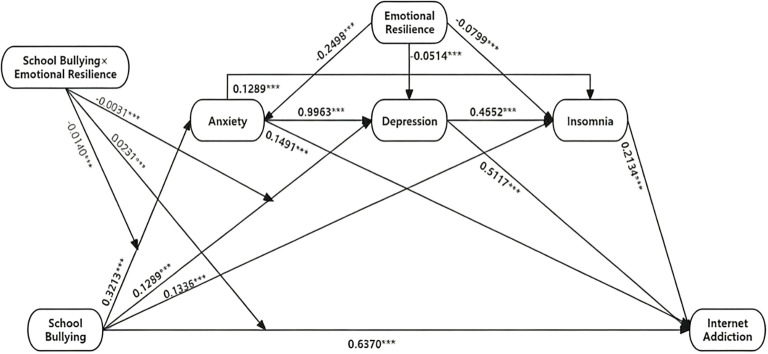
Moderated mediation analysis with emotional resilience as the moderating variable (model 85). ***p < 0.001.

As shown in **Figure 1**, the moderated mediation index of the “X→M3→Y” pathway was -0.0001(95% CI [-0.0008, 0.0006], with the confidence interval containing 0), indicating that this pathway was not significantly moderated by emotional resilience. In contrast, the remaining six pathways passed the moderated mediation effect test: the moderated mediation indices of the “X→M1→Y”, “X→M2→Y”, “X→M1→M2→Y”, “X→M2→M3→Y”, and “X→M1→M2→M3→Y” pathways were -0.0021, -0.0031, -0.0064, -0.0006, -0.0012 respectively, and their corresponding 95% confidence intervals did not contain 0, suggesting significant moderated mediation effects. The moderated mediation index of the “X→M1→M3→Y” pathway was -0.0001(-0.0002, 0), showing a marginal moderating characteristic.

This large-sample cross-sectional study considered both statistical significance and practical effect size. The model exhibited acceptable explanatory power across all variables, with R² ranging from 0.303 to 0.766 and the strongest predictive capacity for depressive symptoms. Small-to-moderate direct correlations existed between school bullying, emotional resilience, depressive symptoms and internet addiction, with reliable statistical and practical value. The interaction of school bullying and emotional resilience produced slight yet significant incremental variance in predicting anxiety, depression and internet addiction, whereas no meaningful moderating effect was detected for insomnia. Although the interaction effect sizes were modest, they were statistically stable and theoretically consistent, which supports the stress-buffering function of emotional resilience in adolescent mental and behavioral outcomes. All effect magnitudes fell within the conventional range of social science research, further confirming the rationality and explanatory adequacy of the model.

Further simple slope analysis confirmed that individuals with high emotional resilience can indirectly buffer the negative impact of school bullying on internet addiction by weakening the transmission effect of school bullying on anxiety, depression, and insomnia.

### 3.6 Results of robustness analysis

To examine the reliability and stability of the research findings, this study conducted robustness tests from two aspects. First, internet addiction was converted into a binary variable (1 = having internet addiction, 0 = without internet addiction) for binary logistic regression analysis. The model R² was 0.163, which explained 16.3% of the variance in internet addiction with good model fit. School bullying, anxiety, depression and insomnia were significantly positively correlated with internet addiction, and emotional resilience showed an obvious protective role. The association direction and statistical significance of all core variables remained stable, which preliminarily confirmed the reliability of the relationships among study variables. Second, based on the original moderated serial mediation model, the path order of the mediating variables of anxiety, depression and insomnia was rearranged, and Model 85 was refitted. The results showed that the association direction, significance level and effect size of core variables were highly consistent with those of the baseline model. Combined with the results of binary logistic regression and the re-estimation by adjusting the order of mediating variables, the core research outcomes showed no obvious changes. It is indicated that the constructed model and moderated serial mediation mechanism have good robustness, and the research conclusions are not affected by the measurement form of the dependent variable or the arrangement order of mediating variables.

## 4 Discussion

### 4.1 Direct effect of school bullying on internet addiction

School bullying counts among the key stressors in adolescents’ growth, and its connection to internet addiction has drawn sustained attention in social psychology and public health. Adolescents who experience school bullying frequently grapple with real-life troubles: strained peer bonds, a lingering lack of security, and low self-worth. The internet, with its built-in anonymity, autonomy and instant feedback, happens to offer them three key compensatory functions. For one thing, it lets them escape real-life anguish. By immersing themselves online, they can temporarily step away from bullying situations, free from the power imbalances they face in daily life. For another, the Internet can also give some emotional support to these teenagers. In virtual communities, they can seek out “like-minded groups”—finding peers with similar experiences to gain emotional understanding. Third, Internet gives them another chance to restore their lost sense of self-worth. Achievements such as winning online games or getting likes on social platforms help mend the self-esteem eroded by bullying. With time going by, this kind of compensation for internet usage will turn into pathological dependence on the internet, forming a vicious cycle in the end: School bullying leads to all sorts of troubles and pains in real life; adolescents’ unfulfilled psychological needs make them lean towards relying even more on the Internet; long-term overuse of the Internet further solidifies these addictive behaviors (32).

In this study, the direct effect size between school bullying and internet addiction aligns with existing research, further validating the stable positive link between them. Even with variations in sample characteristics, measurement tools, or research contexts across studies, the association of school bullying with internet addiction remains within a relatively consistent range. This outcome not only provides new empirical support for the potential statistical association between the two but also confirms that as a risk factor for internet addiction, school bullying exhibits cross-context stability in its correlations—it is not restricted by specific samples or research designs. Moreover, it offers targeted insights for subsequent interventions on adolescent internet addiction, highlighting the need to prioritize the potential association of school bullying experiences with adolescents’ online behaviors. Lower prevalence of school bullying is accordingly correlated with a lower likelihood of internet addiction.

### 4.2 Chained mediation role of depression, anxiety, and insomnia

The findings of this study indicate that anxiety, depression, and insomnia not only serve as independent mediators between school bullying and Internet addiction, but also jointly form a chained mediating pathway. This outcome enriches the framework of the associative pathway linking school bullying to Internet addiction.

Among these psychological and physiological factors, anxiety plays a prominent explanatory role in the association between bullying victimization and internet addictive behaviors. Anxiety is primarily characterized by excessive worry about real-world social situations and extreme sensitivity to negative evaluations. This tends to make adolescents regard face-to-face interactions as high-risk environments and develop strong avoidance tendencies. The anonymity of cyberspace, along with the controllable rhythm of online communication, may thus serve as a “safe buffer” linked to reduced anxiety. The short-term emotional relief associated with online interactions further corresponds to strengthened tendencies to escape reality and rely on the internet. Over time, this temporary emotional coping method is likely to gradually correlate with the emergence of uncontrollable addictive behaviors (33). In comparison, the anhedonia and feelings of helplessness associated with depression mostly lead individuals to passively rely on the internet, merely to fill an emotional void with no clear behavioral motives, which further explains the inner connection between depressive mood and online behavioral dependence. As an important physiological manifestation of psychological stress, insomnia also acts as an indispensable intermediate pathway in the predictive association between school bullying and internet addiction. Persistent psychological stress related to school bullying is associated with anxiety and cognitive rumination, which in turn are linked to sleep disturbances. Long-term sleep deprivation impairs the cognitive control function of the prefrontal cortex, reducing individuals’ self-control over internet use. This ultimately forms the pathway: “school bullying→insomnia→Internet addiction” (34).

Regarding the chain mediating pathway, the logical progression of school bullying→depression/anxiety→insomnia→internet addiction aligns well with the theoretical framework of “stressor -emotion dysregulation -physical disturbance -addiction”. Unlike most previous studies that tend to explore these psychological and behavioral outcomes in isolation, this theoretical interpretation integrates emotional and sleep-related responses into a complete sequential pathway. It illustrates that the adverse impacts of school bullying are not limited to single emotional discomfort, but arise from the overlapping and interactive effects of psychological, physiological and behavioral factors. From a practical perspective, interventions targeting any key link in this developmental chain can effectively reduce the risk of internet addiction among adolescents exposed to bullying. Accordingly, prevention and intervention work should attach equal importance to emotional psychological counseling and sleep health management, so as to interrupt the progressive addictive trajectory at an early stage.

### 4.3 The moderating role of emotional resilience

Findings from this study revealed distinct interactive patterns between emotional resilience and the associations linking school bullying to anxiety, depression, insomnia, and internet addiction. Rather than simply replicating prior conclusions, this interpretation extends the stress-buffering framework to the full relational chain of bullying, emotional disturbance, sleep problems and addictive behaviors (35). Consistent with existing evidence, this study supports the protective role of emotional resilience in bullying-related mental health outcomes. Unlike most previous studies that mainly focused on independent single-pathway moderation, the present study further confirms that emotional resilience functions across a complete serial mediating chain. Moreover, the non-significant moderating effect observed for the insomnia pathway also reveals nuanced differences compared with prior research, providing supplementary evidence for the boundary condition of resilience buffering.

Emotional resilience serves a key regulatory function in buffering against adverse experiences and maintaining psychological stability. Adolescents with higher emotional resilience tend to use adaptive strategies such as cognitive reappraisal and problem-solving, which help reduce the internalization of negative emotions and thereby enhance psychological tolerance and adaptability (36).

From a practical perspective, these results also offer clear implications for school psychological counseling and clinical prevention. Cultivating emotional resilience among vulnerable adolescents can effectively weaken the cascading harmful effects of bullying, and thus reduce the risk of subsequent emotional disorders, sleep disturbance and internet addiction.

### 4.4 Limitations and future directions

First, this study is subject to inherent limitations in sampling design and measurement methods. We used convenience cluster sampling to recruit participants, which allowed for efficient sample collection but is inevitably prone to sampling bias. All core variables were measured using self-reported questionnaires. Adolescents are in a sensitive period of psychological development, so they might deliberately hide it or not disclose the true situation out of fear of being negatively judged by people around them or secondary harm, resulting in information bias. Additionally, the questionnaires cover a broad range of dimensions and include a relatively large number of items, during the long time of questionnaire filling process, respondents will feel mentally tired, and this will decrease the preciseness and strictness of their answers and affect the credibility of research data.

Second, the cross-sectional design has inherent limitations that restrict the inference of causal relationships and dynamic mechanisms among the study variables. Although this study adopted a large sample size to ensure adequate statistical power, data were collected only at a single time point. Consequently, we can only observe static correlations among the core variables, and cannot determine the temporal ordering or causal sequence underlying the chained mediating pathways. A one-time cross-sectional survey is unable to capture the long-term dynamic interplay between school bullying, anxiety, depression, insomnia, and internet addiction. Future research should adopt longitudinal or time-series designs to further verify the causal paths and dynamic relationships observed in this study.

Third, this study only measured bullying victimization without assessing bullying perpetration, so we could not distinguish participants into victims, perpetrators, or bully-victims. As a cross-sectional survey at a single time point, it cannot track subsequent changes in bullying experience or rule out ongoing bullying among adolescents. Future research should simultaneously measure different bullying roles and adopt longitudinal designs to verify relevant associations.

## 5 Conclusions

The findings of this study confirm that school bullying serves as a critical risk factor in the development of psychological and behavioral problems among adolescents. It’s not only about a single direct correlation on its association with adolescent internet addiction; on one hand, it is directly correlated with a higher possibility of adolescents suffering from internet addiction; but on the other hand, it is indirectly linked to internet addiction along with other psychological and physical issues like anxiety, depression and sleeplessness in adolescents. In the whole associative path, emotional resilience plays an obvious moderating function: It could greatly weaken the link of school bullying with adolescent anxiety and depression, so as to lessen the negative psychology of adolescents who have been bullied. The moderated mediational model created in this study is validated by the data: school bullying is not just directly and positively associated with adolescents’ internet addiction, but it is also correlated with the development of adolescent internet addiction via independent mediating effect as well as chained mediating effects of depression, anxiety and insomnia respectively. Through controlling the intensity of the connection between school bullying and bad feelings like sadness and worry, emotional endurance can help reduce the extent to which school bullying relates to negative emotional experiences and provides important mental support for adolescents, avoiding the deterioration of emotional status and subsequent problematic online behaviors, especially excessive internet use. Although similar links among bullying, emotional problems, and internet addiction have been examined in Western studies, this study extends the literature by testing these pathways among Chinese adolescents. The large sample from a urban city in China enhances the applicability of findings to similar adolescent groups, offering empirical support for local mental health and bullying intervention practices.

This is specific and definite help to make all kinds of treatment plans for adolescence’s mentality, as well as prevention and cure of the addiction to Internet. Firstly, it is essential to reinforce the origin prevention and control over school bullying, create a well-functioning system of governance for campus bullying problems, cut down on young people’s chance to get hurt by bullying from the very start, in order to disrupt the starting phase in turning school bullies into anxious, depressed or insomniac individuals. Secondly, it is necessary to focus on the intermediary transmission effect of anxiety, depression and insomnia, take precise and targeted interventions on adolescents who have been bullied, and drive the adoption of a two-pronged intervention strategy of emotion management and enhancement of sleep health. Through cognitive-behavioral therapy, sleep hygiene guidance, and other approaches, we can interrupt the developmental chain where emotional issues and sleep disorders lead to Internet addiction. Also pay attention to the importance of emotional resilience as a moderator and adopt some ways to increase adolescents’ sensitivity to negative emotions, improve the elasticity of their own emotional regulation ability, and enhance their psychological cushioning strength when encountering school bullies and other bad things. Carry out school-based emotional regulation course training, organize group psychological counseling to carry out individual psychological consultation, which can reduce the transmission rate of each path from the regulation level, and form a robust psychological protection wall for adolescents. The above mentioned intervention suggestions form a solid theoretical and empirical foundation on which we could construct an adolescent mental health support system in the digital age, they also serve as operational reference for schools, parents and related governmental departments who need to make policy decisions about adolescent internet addiction prevention and mental care.

In summary, as a core risk factor in adolescent development, school bullying is closely linked to internet addiction in adolescents through mediating associations with psychopathological symptoms such as anxiety, depression and insomnia, and emotional resilience can effectively weaken the intensity of this associative chain’s formation.

## Data Availability

The original contributions presented in the study are included in the article/supplementary material. Further inquiries can be directed to the corresponding authors.

## References

[1] United Nations Educational, Scientific and Cultural Organization (UNESCO) Institute of School Violence and Prevention, Ewha Womans University . School violence and bullying: Global status report. Paris, France: UNESCO (2017). Available online at: https://unesdoc.unesco.org/ark:/48223/pf0000368092 (Accessed May 25, 2026).

[2] Daley SF Wasim M Nickerson AB . Identifying and addressing bullying behaviors. In: StatPearls. Treasure Island, FL: StatPearls Publishing (2026). Available online at: https://www.ncbi.nlm.nih.gov/books/NBK441930/ (Accessed May 25, 2026). 28722959

[3] United Nations Educational, Scientific and Cultural Organization (UNESCO) . School violence and bullying: Global status and trends, drivers and consequences. Paris: Author (2018).

[4] Yuan XF Zhang XL . Progress of school bullying research in China: Analysis of the knowledge map on basis of CiteSpace. Ludong Univ J (Philosophy Soc Sci Edition). (2024) 41:48–55. doi: 10.20063/j.cnki.CN37-1452/C.2024.04.008

[5] Bitar Z Elias MB Malaeb D Hallit S Obeid S . Is cyberbullying perpetration associated with anxiety, depression and suicidal ideation among Lebanese adolescents? Results from a cross-sectional study. BMC Psychol. (2023) 11:53. doi: 10.1186/s40359-023-01091-9 36829238 PMC9951827

[6] Dou Y Wongpakaran T Wongpakaran N O'Donnell R Bunyachatakul S Pojanapotha P . Bullying victimization moderates the association between social skills and self-esteem among adolescents: A cross-sectional study in international schools. Children. (2022) 9:1606. doi: 10.3390/children9111606 36360334 PMC9688646

[7] Evans CBR Smokowski PR . Theoretical explanations for bullying in school: How ecological processes propagate perpetration and victimization. Child Adolesc Soc Work J. (2016) 33:365–75. doi: 10.1007/s10560-015-0432-2 30311153

[8] Cohen S Wills TA . Stress, social support, and the buffering hypothesis. psychol Bull. (1985) 98:310–57. doi: 10.1037/0033-2909.98.2.310 3901065

[9] Yu L Wu P Zhang Y Zhao X Qu S Gao C . The relationship between traditional and cyber bullying victimization and mobile phone addiction among adolescents: The mediating role of friendship quality and depression. psychol Rep. (2025). doi: 10.1177/00332941251383487

[10] Kardefelt-Winther D . A conceptual and methodological critique of internet addiction research: Towards a model of compensatory internet use. Comput Hum Behav. (2014) 31:351–4. doi: 10.1016/j.chb.2013.10.059 38826717

[11] Alavi SS Ferdosi M Jannatifard F Eslami M Alaghemandan H Setare M . Behavioral addiction versus substance addiction: Correspondence of psychiatric and psychological views. Int J Prev Med. (2012) 3:290–4. doi: 10.4103/0974-5999.98240 PMC3354400 22624087

[12] Billieux J Maurage P Lopez-Fernandez O Kuss DJ Griffiths MD . Can disordered mobile phone use be considered a behavioral addiction? An update on current evidence and a comprehensive model for future research. Curr Addict Rep. (2015) 2:156–62. doi: 10.1007/s40429-015-0054-y 30311153

[13] Lu X Watanabe J Liu Q Uji M Shono M Kitamura T . Internet and mobile phone text messaging dependency: Factor structure and correlates among Japanese adults. Comput Hum Behav. (2011) 27:1702–9. doi: 10.1016/j.chb.2011.02.009

[14] Brand M Young KS Laier C Wölfling K Potenza MN . Integrating psychological and neurobiological considerations regarding the development and maintenance of specific Internet-use disorders: The interacting person-affect-cognition-execution (I-PACE) model. Neurosci Biobehav Rev. (2016) 71:252–66. doi: 10.1016/j.neubiorev.2016.08.033 27590829

[15] Zhu Z Sang B Liu J Zhao Y Liu Y . The association between emotional resilience and mental health among Chinese adolescents in school settings: The mediating role of positive emotions. Behav Sci. (2025) 15:567. doi: 10.3390/bs15050567 40426345 PMC12108795

[16] Korkmaz Z Çiçek Ç Buluş M Şanlı ME Yıldırım M . The role of resilience in the relationship between cyberbullying and depression, anxiety, and stress among adolescents. Brain Behav. (2025) 15:e70916. doi: 10.1002/brb3.70916 41116652 PMC12537845

[17] Jing J Luo J Hou X Yan Y . The effect of cyberbullying perpetration on depression among adolescents in western China: The mediating role of self-control and online gaming addiction and the moderating role of meaning in life. Front Psychol. (2026) 17:1749786. doi: 10.3389/fpsyg.2026.1749786 41890950 PMC13013446

[18] Li L Cai J Wang C Mu YF Deng ZY Deng AP . The association between school bullying and internet addiction among adolescents: A moderated mediation model. Front Public Health. (2025) 13:1502726. doi: 10.3389/fpubh.2025.1502726 40213425 PMC11983463

[19] Xie Y Zhang MM Wang C Cai J Wang Y Mu YF . The effect of bullying victimization on internet addiction: Mediated by cyberbullying perpetration and moderated by social support. BMC Public Health. (2025) 25:2856. doi: 10.1186/s12889-025-23905-8 40836288 PMC12366385

[20] Raudenbush SW Bryk AS . Hierarchical linear models: Applications and data analysis methods. Thousand Oaks, CA: Sage Publications. (2002).

[21] Sun JL Liu HH . The development of the bullying behavior scale for adolescents. Stud Psychol Behav. (2022) 20:255–60. doi: 10.12139/j.1672-0628.2022.02.016

[22] Spitzer RL Kroenke K Williams JBW Löwe B . A brief measure for assessing generalized anxiety disorder: The GAD-7. Arch Internal Med. (2006) 166:1092–7. doi: 10.1001/archinte.166.10.1092 16717171

[23] Spitzer RL Kroenke K Williams JB . The PHQ-9: Validity of a brief depression severity measure. J Gen Internal Med. (2001) 16:606–13. doi: 10.21706/pdp-23-3-193 PMC1495268 11556941

[24] Morin CM . Insomnia: Psychological assessment and management. New York, NY: Guilford Press (1993). Available online at: http://catdir.loc.gov/catdir/bios/guilford051/93006564.html (Accessed May 31, 2026).

[25] Young KS . Internet addiction test (IAT) manual. Bradford, PA: Center for Internet Addiction Recovery (1998).

[26] Connor KM Davidson JRT . Development of a new resilience scale: The Connor-Davidson Resilience Scale (CD-RISC). Depression Anxiety. (2003) 18:76–82. doi: 10.1002/da.10113 12964174

[27] Zhang M Lu J . The development of adolescents' emotional resilience questionnaire. J psychol Sci. (2010) 33:24–7.

[28] Podsakoff PM MacKenzie SB Lee JY Podsakoff NP . Common method biases in behavioral research: A critical review of the literature and recommended remedies. J Appl Psychol. (2003) 88:879–903. doi: 10.1037/0021-9010.88.5.879 14516251

[29] Hair JF Black WC Babin BJ Anderson RE . Multivariate data analysis. Boston, MA: Cengage (2019). Available online at: https://www.cengage.com/c/ebook-multivariate-data-analysis-8e-hair-babin-anderson/9781473756557/ (Accessed May 31, 2026).

[30] Han ZY Ye ZY Zhong BL . School bullying and mental health among adolescents: A narrative review. Trans Pediatr. (2025) 14:463–72. doi: 10.21037/tp-2024-512 40225076 PMC11982999

[31] Park A . The roles of anxiety and depressive symptoms in the relationship between school bullying victimization and suicidal ideation among South Korean college students: A serial multiple mediation model. Int J Environ Res Public Health. (2025) 22:256. doi: 10.3390/ijerph22020256 40003482 PMC11855795

[32] Bronfenbrenner U . The ecology of human development: Experiments by nature and design. Cambridge, Massachusetts: Harvard University Press (1979).

[33] Wang J Wang N Qi T Liu Y Guo Z . The central mediating effect of inhibitory control and negative emotion on the relationship between bullying victimization and social network site addiction in adolescents. Front Psychol. (2025) 15:1520404. doi: 10.3389/fpsyg.2024.1520404 40242396 PMC12002087

[34] Jiang S Ding JQ Liu Y Lu YY Li XQ Chen J . The effect of cyber-bullying/cyber-victimization on sleep quality in early adolescence: A serial mediation model of social anxiety and depression mood. psychol Dev Educ. (2023) 39:85–96. doi: 10.16187/j.cnki.issn1001-4918.2023.01.10

[35] Lin LY Chien YN Chen YH Wu CY Chiou HY . Bullying experiences, depression, and the moderating role of resilience among adolescents. Front Public Health. (2022) 10:872100. doi: 10.3389/fpubh.2022.872100 35692326 PMC9174695

[36] Han F Duan R Huang B Wang Q . Psychological resilience and cognitive reappraisal mediate the effects of coping style on the mental health of children. Front Psychol. (2023) 14:1110642. doi: 10.3389/fpsyg.2023.1110642 37077843 PMC10106575

